# Genome-Wide Identification and Characterization of RBR Ubiquitin Ligase Genes in Soybean

**DOI:** 10.1371/journal.pone.0087282

**Published:** 2014-01-28

**Authors:** Pei Chen, Xiaolian Zhang, Tuanjie Zhao, Yan Li, Junyi Gai

**Affiliations:** Soybean Research Institute, National Center for Soybean Improvement, MOA Key Laboratory for Biology and Genetic Improvement of Soybean (General), National Key Laboratory of Crop Genetics and Germplasm Enhancement, Nanjing Agricultural University, Nanjing, Jiangsu, China; Nazarbayev University, Kazakhstan

## Abstract

RBR (RING1-IBR-RING2) proteins play an important role in protein ubiquitination and are involved in many cellular processes. Recent studies showed plant RBR genes were induced by abiotic and biotic stresses. However, detailed studies on RBR genes in the important oil crop, soybean (*Glycine max* (L.) Merr.), is still lacking. Here we performed a genome-wide search and identified 24 RBR domain-containing genes from the soybean genome sequence and cloned 11 of them. Most soybean RBR proteins contain a highly conserved RBR supra-domain. Phylogenetic analyses indicated all 24 soybean RBR proteins are most related to the RBR proteins from *Phaseolus vulgaris*, and could be classified into seven groups including Ariadne A, Ariadne B, ARA54, Plant IIA, Plant IIB, Plant IIC, and Helicase. Tandem duplication and block duplication were found among the Ariadne B and Plant IIC group of soybean RBR genes. Despite the conserved RBR supra-domain, there are extensive variations in the additional protein motifs and exon-intron structures between different groups, which indicate they might have diverse functions. Most soybean RBR proteins are predicted to localize in nucleus, and four of them were experimentally confirmed by GFP fusion proteins. Soybean RBR genes are broadly expressed in many tissue types with a little more abundant in the roots and flowers than leaves, stems, and seeds. The expression of *GmRTRTP3* (Plant IIB) and *GmRTRTP5* (Plant IIC) are induced by NaCl treatment, which suggests these RBR genes might be involved in soybean response to abiotic stresses.

## Introduction

In eukaryotes, protein ubiquitination is a key biochemical mechanism and play a fundamental role in multiple cellular processes, such as protein homeostasis [1], signal transduction [2]–[5], development [6], [7], differentiations and programmed cell death (PCD) [2]. During ubiquitination process, ubiquitin is transferred to a target protein through a trio of enzymes: ubiquitin activating enzymes (E1), which activate the ubiquitin by an ATP dependent manner; ubiquitin-conjugating enzymes (E2 or Ubc), which has two jobs in the conjugation cascade: to accept the ubiquitin protein from the E1 enzyme, and to pass it to a downstream protein such as ubiquitin protein ligases (E3); E3s, which have the highest diversity, interact directly with the target proteins and are responsible for the specificity of ubiquitination. E3s mediate the attachment of ubiquitin to a lysine (Lys) ε-amino group on the target protein. The diversity of E3s have been shown in species whose genome has been completely sequenced such as *Arabidopsis*, in which there are more than one thousand and four hundred predicted E3s [6].

Recently, a new E3 protein family involved in ubiquitination, which is a subset of RING (Really Interesting New Gene) proteins,is characterized by the presence of a RING1-IBR-RING2 (RBR) supra-domain. This tripartite domain is also known as a TRIAD (for two RING fingers and a DRIL) [8], which is composed of three consecutive domains including a N-terminal RING1, an IBR (In Between Ring), and a C-terminal RING2 domain. The N-terminal RING1 is a canonical RING finger with a C3HC4 signature of conserved cysteine and histidine residues, which has a general formula C–X_2_–C–X_(10–24)_ –C–X_(1–6)_–H–X_2_–C–X_2_–C–X_(14–25)_–C–X_(2–9)_–C. The IBR domain has a typical C6HC signature with a shorter and strict formula: C–X_2_–C–X_(9–11)_–C–X–H– X_2_–C–X_(1–4)_–C–X_4_–C–X_2_–C, which is found only in RBR proteins [9], [10]. The C-terminal domain, often called as RING2, resembles a much shorter RING finger with C3HC4 signature.

Over the past decades, a large and diverse group of proteins is characterized by containing the RBR supra-domain, and RBR proteins have been found in all eukaryotic organisms [5], [6]. Based on the conserved RBR domain sequence, RBRs can be divided into 14 subfamilies, of which 11 subfamilies are present in animals [8], [9], [11] and only four subfamilies in plants (Ariadne, ARA54, Helicase, and Plant II) [12].

Accumulating evidence has suggested that RBR proteins play an important role in degradation of abnormal protein and most short-lived regulatory protein, mitochondria dynamic changes, apoptosis and indirectly regulation of transcription in human and animals [10], [13]–[16]. The best-known member of RBR protein is PARKIN in human, a member of Parkin subfamily. The mutations in the *parkin* gene have been found in cases of juvenile, autosomal recessive familial Parkinson disease [17]. Some genes of Ariadne subfamily have been shown to function as interaction partners of ubiquitin-conjugating enzymes (E2s) in fruitfly (*Drosophila*), *C. elegans* and humans [14], [18], [19]. However, the functions of RBR proteins in plants remain largely unknown. Recently, studies have implicated some RBR proteins in Arabidopsis were induced by abiotic and biotic stresses such as virus, cold, heat, salt, and ultraviolet (UV) stimuli [12]. A member of the Ariadne subfamily of RBR in Arabidopsis, AtARI12, was shown as a downstream target of two bZIP transcription factors, HY5 and HYH, which were two key players mediating light-regulated developmental and physiological changes [20]. The *HpARI* gene from *Hypericum perforatum*, another Ariadne gene, was involved in the apospory and response to UV-B. The *HAPPY* (*Hypericum APOSPORY*) locus containing a truncated *HpARI* gene was associated with apospory [21]. To our knowledge, there are no reports on the functions of other plant RBR subfamilies members except Ariadne.

Although some RBR genes have been functionally characterized in human and animals, the functions of the majority of RBR members remain unknown. Especially in soybean (*Glycine max* (L.) Merr.), there are no reports on detailed characterization of RBR proteins in this important oil crop. In this study, we performed a genome-wide search for RBR genes in soybean. Twenty-four RBR domain-containing genes were identified from the soybean genome sequence (http://www.phytozome.net/soybean). We identified the conserved RBR supra-domain from these 24 soybean RBR proteins, and analyzed the conservations and diversifications of these protein motifs. We did comprehensive phylogenetic analyses of 650 RBR protein sequences from 32 plant species including the 24 soybean RBR proteins, and a subset of RBR proteins from three legume species including *Glycine max*, *Medicago truncatula* and *Phaseolus vulgaris*. The gene duplication patterns were analyzed to investigate the evolutionary origin of these RBR genes. The sub-cellular locations of the soybean RBR proteins were predicted and four of them were experimentally confirmed by GFP fusion proteins. The gene expression pattern of soybean RBR genes was investigated by published data and RT-PCR/qRT-PCR in this study. Our results would provide valuable information to further investigate the functions of RBR proteins in soybean, an important oil crop worldwide.

## Materials and Methods

### Bioinformatic Analysis

To identify the RBR genes in soybean, BLASTP was performed using the putative *Arabidopsis* and *Oryza sativa* RBR sequences as seeds to search in soybean genome sequence database (http://www.phytozome.net/soybean). Protein sequences were evaluated using Pfam (http://pfam.sanger.ac.uk/) and SMART (http://smart.embl-heidelberg.de/), and the sequences having no RBR domains or containing less than 180 residues were discarded.

The sequences were aligned using CLUSTALX2.1 and the alignments were edited with the GeneDoc 2.7 sequence editor. Maximum Likelihood fits of 48 different amino acid substitution models were tested using MEGA5 [22], and models with the lowest BIC (Bayesian Information Criterion) scores are considered to best describe the substitution pattern and the parameters were used for the phylogenetic tree construction. Maximum-likelihood (ML) trees were obtained using PhyML (approximate likelihood ratios analysis) [23]. The neighbor-joining (NJ) trees were constructed in MEGA5. All phylogenetic analyses had been done with above two methods of ML and NJ with 1000 bootstraps. The dendrograms were edited with FigTree (http://tree.bio.ed.ac.uk/software/figtree/).

The duplication patterns of the soybean RBR genes were analyzed based on their gene locations in the soybean genome. Tandem duplicated genes are located next to each other, and block duplications were detected with Synteny plot in Plaza (http://bioinformatics.psb.ugent.be/plaza/synteny). First, the RBR genes were analyzed with BLAST in Plaza, and then the gene or gene family IDs were putted into Synteny plot to detect the duplication patterns.

The protein domains and conserved motif were analyzed using pfam (http://pfam.sanger.ac.uk/), SMART (http://smart.embl-heidelberg.de/) and MEME (http://meme.nbcr.net/meme/). Coiled-coil domains in these RBR domain-containing protein sequences were predicted with MARCOIL [24], and COILS [25].

### Soybean Materials and Treatments

Soybean [Glycine max (L.) Merr.] var. Nannong 1138-2 was used throughout the experiments described here, and the seeds were provided by the National Center for Soybean Improvement (Nanjing, China). Soybean seeds were germinated in clean sand in a growth chamber with a photoperiod of 16/8 h (day/night), day/night temperature of 28/25°C, and a relative humidity of 70%. After germination, the seedlings were transferred to a hydroponic system [26] and grown in a glass-room under the same condition as described above. Young leaves, stems, and roots were collected from 4-week-old seedlings (V2 stage); blooming flowers were sampled from plants at R2 stage, whereas developing seeds and pods were collected since the beginning of R3 stage to R7 stage, at five days intervals. Salt stress treatment (200 mM NaCl) was performed on the tenth days after seedlings were transferring into hydroponic system. The roots were harvested at 0, 0.5, 1, 2, 4, 6, 8, 12, 48 and 72 h after salt treatment, immediately frozen with liquid N_2_ and stored at −80°C. Two independent experiments were performed.

### Isolation of Soybean RBR Genes

Total RNA was isolated with Trizol according to the manufacturer’s protocol (Invitrogen). Approximately, 0.2 µg of mRNA was used to synthesize the cDNA using a cDNA synthesis kit (TaKaRa) following the manufacturer’s protocol. The cDNA sequences of soybean RBR proteins were amplified in a 50 µl reaction volume containing 20 pmol of each forward and reverse primers,0.8 mM dNTPs, 2.0 U PrimeSTAR™ HS DNA polymerase (TaKaRa), 2 µl cDNA in 1X buffer, and 1 µl MgCl_2_ (50 mM). PCR conditions were set up according to the polymerase manufacturer’s protocol (TaKaRa) and annealing temperatures of primers (Table S1). PCR amplified DNA fragments were sub-cloned into the pGEM-T vector (Promega) and sequenced at Invitrogen (GenBank IDs were listed in Table S2).

### RT-PCR

The Semi-quantitative RT-PCR was performed in a final volume of 20 µl containing 2 µl of diluted cDNA, 10 µl 2×Premix Taq version 2.0 Mix (TaKaRa), and 200 nM of forward and reverse primers (Table S1). The thermal cycling conditions were as follows: 32 cycles of 95°C for 30 s for denaturation, 30 s for annealing and 45 s for extension, the annealing temperatures is based on the designed primer. Soybean Tubulin B3 (U12286) gene was used as the internal control, and three replicates were performed.

For qRT-PCR analysis, RNA was extracted from roots as mentioned above. cDNA was prepared using the cDNA synthesis kit (TaKaRa) from 200 ng of total RNA. qRT-PCR was performed using the homemade SYBR Green kit (Table S3) using Applied Biosystems FAST 7500 real-time PCR. Expression levels were normalized to Tubulin B3 (U12286). All qRT-PCR experiments were independently performed in two biological replicates and three technical replicates. Technical replicates were averaged first, then relative expression values were calculated [27]. Amplification efficiency of all primers was determined by generation of dilution curves, the plotting of the *Ct* values against the logarithm of the initial concentration (the standard curve method). Primers used in these experiments are listed in Table S1.

### Transient Expression in Arabidopsis Mesophyll Protoplasts

*Arabidopsis* plants (Col-0 ecotype) were grown in a glass room at 8/16 h light/dark short-day at 19/23°C (light/dark) with a relative humidity of 55–75%, after 40 days, the plants was moved to long-day conditions (16/8 h of light/dark) with light illumination provided by high pressure sodium lamps.

Recombinant vectors were constructed using Gateway system according to the manufacture protocol (Invitrogen), the RBR-GFP fusion fragments were sub-cloned into the binary pMDC83 vector under CaMV 35S promoter. The transient expression of RBR-GFP fusion genes using *Arabidopsis* mesophyll protoplasts was performed following Jen Shen’s laboratory protocol [28] and Fu-Hui Wu’s modification [29]. Confocal analyses were performed using an inverted laser scanner confocal microscope (TCS SP2; Leica).

## Results

### Conservation of Soybean RBR Protein Sequences

**Figure 1:**
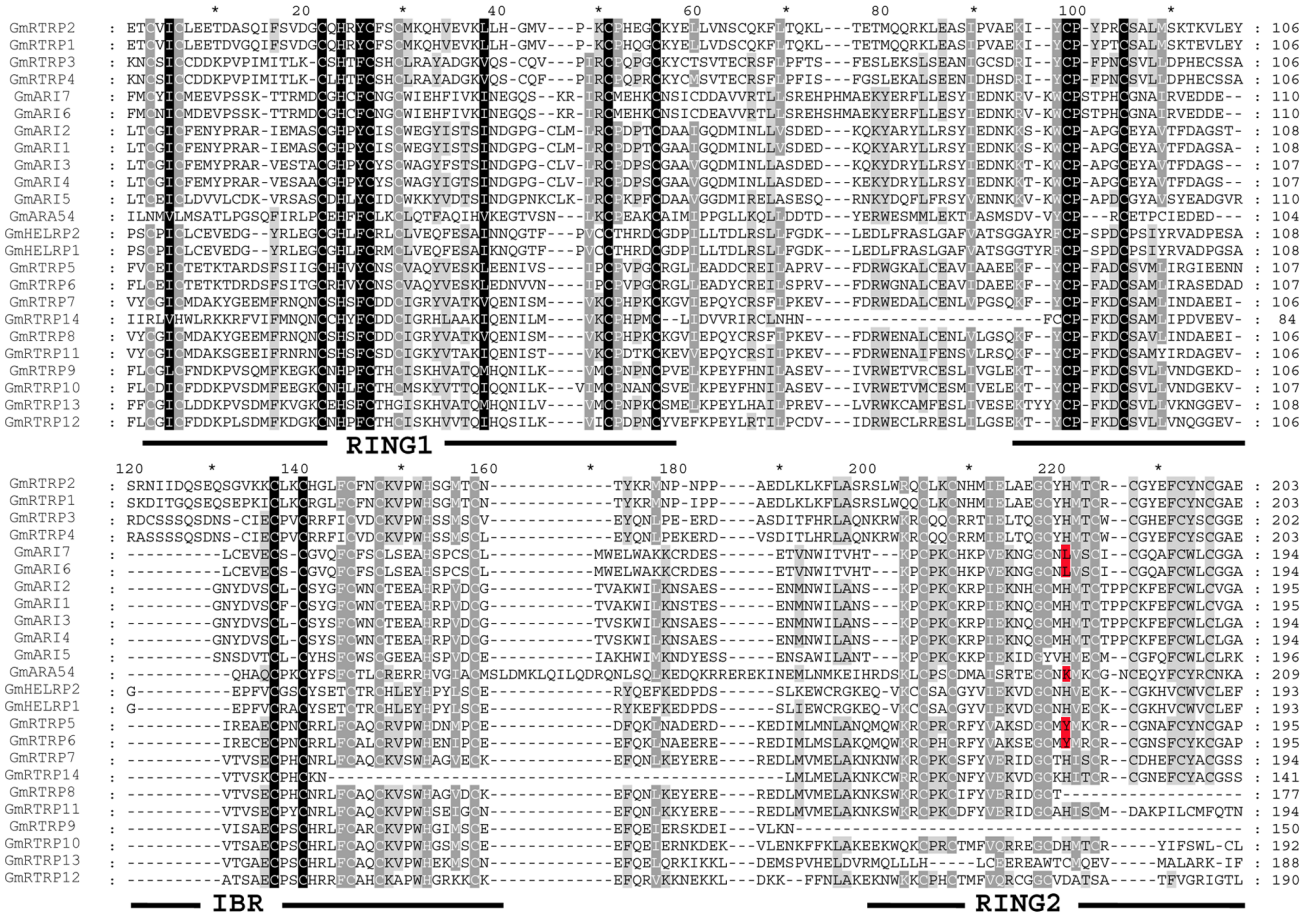
Multiple sequence alignment of Soybean RBR proteins. Black, gray, and light gray shading indicate the identities and similarity among these sequences is 100%, 80%, and 60%, respectively. Hyphens represent gaps, RING1-IBR-RING2 are marked at the bottom of the alignment with solid lines. The first two consevered Cys residues of RING1 of GmARA54 were omitted in this figure due to its extremely long nonconserved sequence between RING1 and RBR. The amino acid residues in red color indicate the substitutions of His residue in RING2 domain.

Using BLASTP search, we identified 24 genes from the soybean whole genome that belong to the RBR protein family (Table 1). Multi-alignment of these 24 RBR protein sequences revealed that 18 of them contained a complete RING1-IBR-RING2 tripartite supra-domain, and six proteins (GmRTRP9-GmRTRP13) had an incomplete RBR domain with different amino acid residue deletions in RING2 (Figure 1). The IBR domain with the signature CP-X_1–5_-C-X_9–17_-C-X_1–2_-C-X_4_-C-X_2_-C-X_4_-H-X_4_-C (C6HC) is conserved in all 24 proteins.

**Table 1:** List of 24 RBR genes in Soybean.

Group^a^	UniGene	ESTs/Full-length cDNA^b^	Amino Acids/kD	pI	Sub cellular location^c^
**ARIDINE A**					
GmARI6	Glyma12g05050	Cloned	580/66.6	5.29	N/N
GmARI7	Glyma11g12920	Cloned	580/66.5	5.62	N/N
**ARIDINE B**					
GmARI1	Glyma11g13750	Cloned	586/67.0	5.26	N/N
GmARI2	Glyma12g05740	Cloned	586/67.1	5.26	N/N
GmARI3	Glyma15g03590	Cloned	589/67.4	5.00	N/N
GmARI4	Glyma13g41830	GMFL02-45-J23	589/67.2	4.92	N/N
GmARI5	Glyma12g03030	−	483/56.6	6.85	n.a/N
**HELECASE** d					
GmHELRP1	Glyma18g01820	Cloned	1729/196.1	6.69	n.a/N
GmHELRP2	Glyma11g37910	AW598065.1	1736/196.7	6.85	n.a/C
**ARA54**					
GmARA54	Glyma09g33900	EV272922.1	583/66.9	5.08	n.a/N
**PLANT IIA** e					
GmRTRP1	Glyma11g15820	Cloned	558/63.8	5.16	N/N
GmRTRP2	Glyma12g07640	Cloned	559/63.9	7.37	N/N
**PLANT IIB**					
GmRTRP3	Glyma09g02340	Cloned	511/58.3	4.48	N/N
GmRTRP4	Glyma15g13240	Cloned	514/58.7	4.45	N/N
**PLANT II C**					
GmRTRP5	Glyma09g08670	Cloned	331/37.3	6.53	N/N
GmRTRP6	Glyma15g20350	−	333/37.6	6.04	N/N
GmRTRP7	Glyma07g04970	−	293/33.8	5.04	n.a/N
GmRTRP8	Glyma07g04990	−	275/31.3	4.53	n.a/N
GmRTRP9	Glyma11g23590	−	158/18.1	7.05	n.a/S
GmRTRP10	Glyma11g23850	−	203/23.8	5.61	n.a/S
GmRTRP11	Glyma16g01530	−	288/33.3	5.03	n.a/N
GmRTRP12	Glyma18g07170	−	222/25.3	8.30	N/N
GmRTRP13	Glyma18g07180	−	255/29.1	7.75	C/C
GmRTRP14	Glyma07g04980	−	265/30.9	9.12	n.a/C

a The group was classified using phylogenetic analysis.

b Hyphens (−) indicate no EST were detected in databases.

c WOLF PSORT/BaCeILo. C, chloroplast; N, nucleus; n.a, not available; S, secretory;

d The genes in HELECASE group were designated as HELRP (Helicase-like RBR Protein);

e The genes in PLANT II group were designated as RTRP (Reverse Transcriptase-like domain-containing RBR Proteins).

The multi-sequence alignment further revealed critical conserved amino acid residues, such as Ile, Phe, Tyr, Leu and Pro in RBR domains (Figure 1). In RING1 domain, the Ile and Phe residues before the second and fifth Cys are conserved among 24 RBR proteins, and a Pro residue follows the seventh Cys. In the IBR signature, there is an extremely conserved Pro residue in the IBR signature after the first Cys residue (Figure 1). All these conserved positions might be important for the protein three-dimensional structures and functions. However, we noticed that five proteins (GmARI7, GmARI6, GmARA54, GmRTRP5 and GmRTRP6) had several mutations in central His position of RING2: His residues were replaced by Leu in GmARI6 and GmARI7, by Lys in GmARA54, and by Tyr in GmRTRP5 and GmRTRP6 (Figure 1).

### Classification of Soybean RBR Proteins

**Figure 2:**
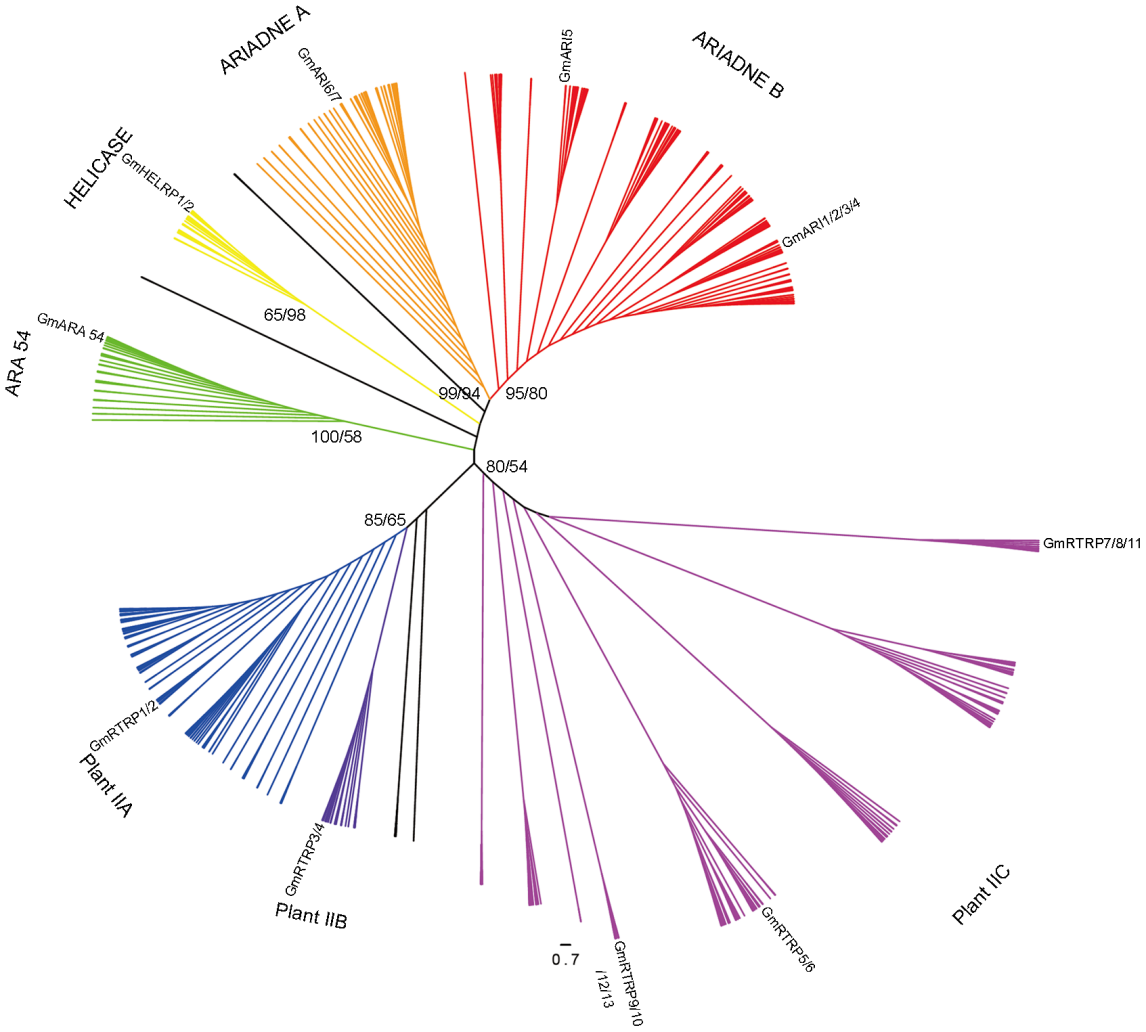
Phylogenetic analysis of 650 plant RBR sequences. Maximum-likelihood (ML) unrooted tree is shown and the main branches that correspond to the seven groups are indicated with different colors. The relative positions of the soybean RBR proteins are labeled with their names, and the numbers at the branch points indicate the bootstrap value of the seven groups (ML/NJ).

Phylogenetic analyses were performed using 650 RBR protein sequences from 32 plant species including the 24 soybean RBR proteins. Due to the different lengths and abundant diversity of the functional domains of the RBR proteins [8], [11], the conserved RBR domain sequences (about 200 amino acids residues in length) were used for the analyses. As shown in Figure 2, all 650 RBR proteins from viridiplantae can be classified into seven groups with the exception of some orphan from Algae and Bryophytes, including Ariadne A, Ariadne B, Helicase, ARA54, Plant IIA, Plant IIB and Plant IIC, which is consistent with the previous study [12]. These seven groups can be further grouped into the four subfamilies (Ariadne, ARA54, Helicase, and Plant II) corresponding to the previous study [12].

The 24 soybean RBR genes are also classified into the seven groups (Figure S1). The Ariadne A and Ariadne B group contain two and five soybean genes, respectively. The Helicase subfamily, which is only found in higher plants, contains two soybean genes: *GmHELRP1* and *GmHELRP2*. The ARA54 subfamily has only one soybean protein, GmARA54. Each of Plant IIA and Plant IIB groups contains two soybean genes, and the group Plant IIC contained ten soybean genes (Table 1).

### Evolutionary Patterns of Soybean RBR Proteins

**Figure 3:**
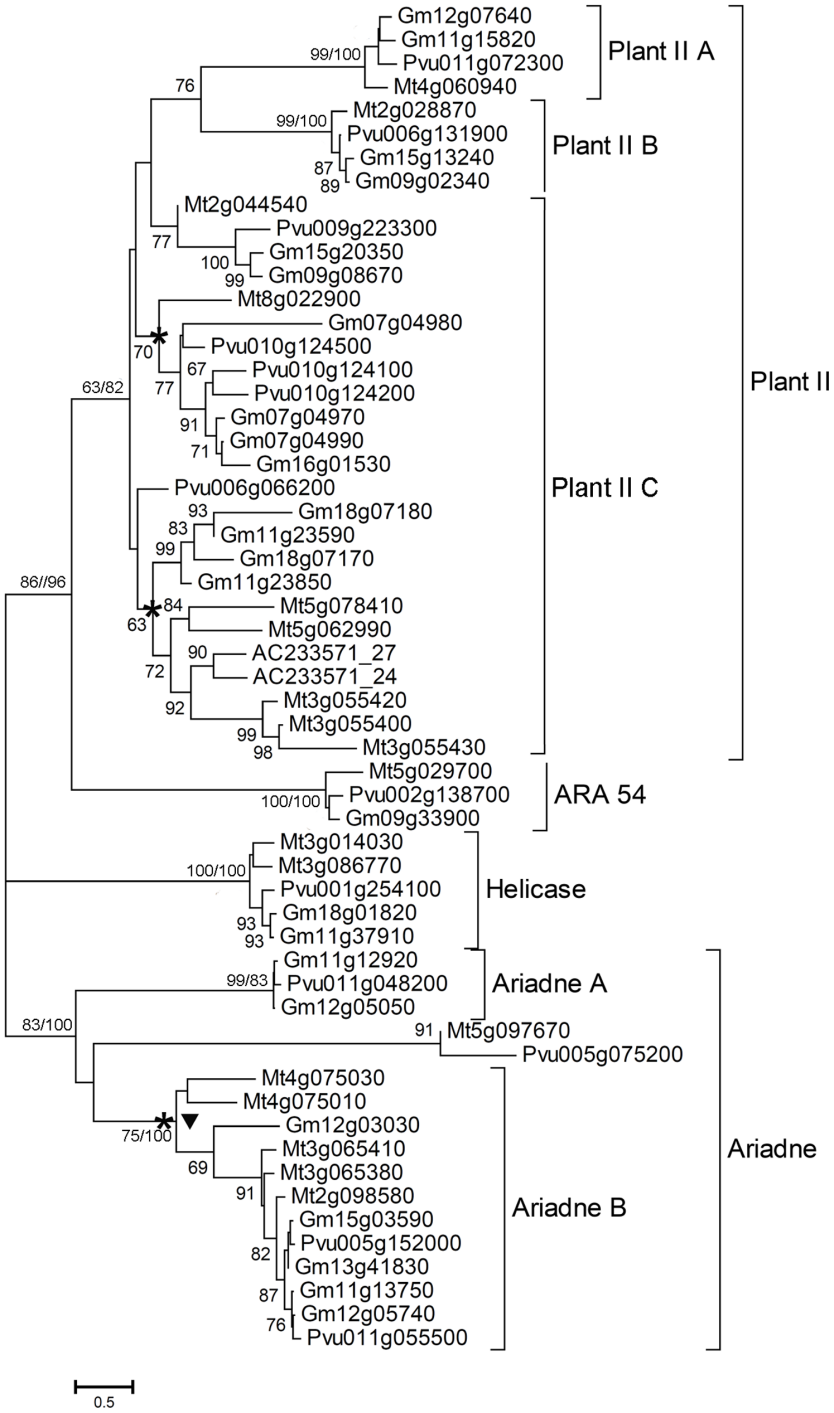
Neighbor-joining (NJ) tree of RBR proteins from *Glycine max* (Gm), *Medicago truncatula* (Mt) and *Phaseolus vulgaris* (Pvu). Numbers refer again to bootstrap support (in percentage). Asterisks indicate the group contains tandem duplication genes, triangle indicates the group has block duplication genes. The numbers at the branch points indicate the bootstrap value of the seven groups (ML/NJ).

*Medicago truncatula* and *Phaseolus vulgaris* are two closely relatives of soybean, and above phylogenetic analysis show that the RBR proteins of these three species of Leguminosae are classed into the same branches (Figure S1). To further investigate the evolutionary patterns of the soybean RBR proteins, the phylogenetic relationship of RBR proteins from *G. max*, *M. truncatula* and *P. vulgaris* was studied. As shown in Figure 3, most soybean RBR proteins are closely related to *P. vulgaris*.

We next analyzed the gene duplication events of these 24 soybean RBR genes and observed both tandem duplication and block duplication patterns. Three tandem repeated genes, *Gm07g04970*, *Gm07g04980*, and *Gm07g04990*, were observed in the group of Plant IIC (Figure S2 A) in *G. max*, deduced from their genomic locations (Figure 3). Tandem duplicated genes were also found in *M. truncatula* (*Mt3g055400*, *Mt3g055420* and *Mt3g055430*) and *P. vulgaris* (*Pvu010124100* and *Pvu010124200*) within Plant IIC group (Figure 3). Gene expansion caused by block duplication was observed in the Ariadne B (Figure S2 B and Figure 3). We did not find obvious gene duplications in the other five groups of RBR proteins in soybean.

### Structural Diversification of Soybean RBR Proteins

**Figure 4:**
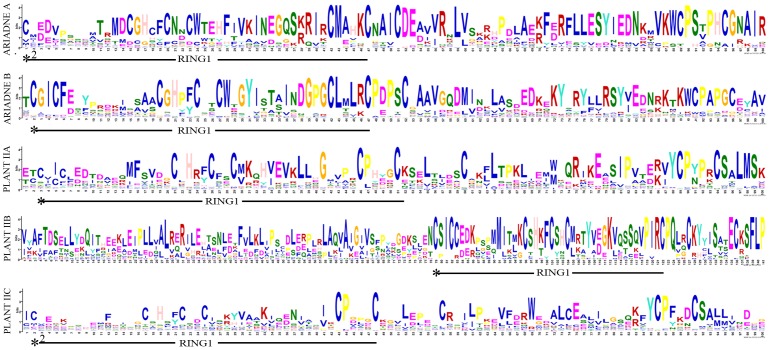
Conservation and diversity of the RING1 motif in RBR domains. The schematic representation of the RING1 motif (indicated by solid lines) in RBR domain is elucidated by MEME. Asterisks indicate the first conserved Cys residues of RBR domain, while asterisks with a superscript number indicate responding conserved Cys residues site. The height of a letter in the Logo indicates its relative frequency at the given position (x -axis) in the motif.

Although the RBR domain contains extremely conserved Cys, His and some other amino acids residues, we notice that the sequences of RBR domain between different groups contain a large number of variations. For example, the sequences of RING1 domain between Ariadne A and Ariadne B have different conserved amino acids residues, especially the residues between the conserved Cys and His (Figure 4), while the IBR and RING2 domains are more conservative compared with RING1(Figure S3). Similarly, the residues between Cys and His in the RBR domain of Plant IIC are quite diverse as compared with Plant IIA and Plant IIB (Figure 4 and Figure S3).

**Figure 5:**
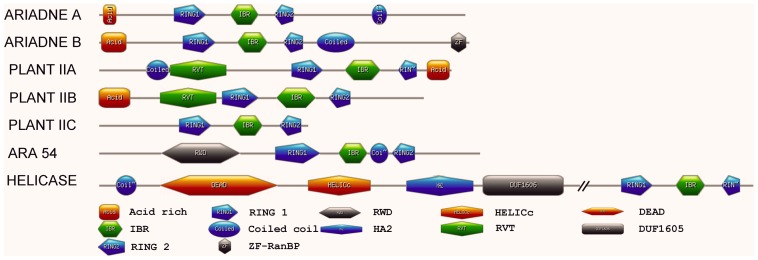
The typical protein structures of different RBR subfamilies. The double-slash in the Helicase-containing protein represents the omitted region due to its much longer sequence than what is shown here.

In addition to the conserved RBR supra-domain, there are other various structural domains in these soybean RBR proteins (Figure 5). Protein sequence analyses using MEME suggest that the protein sequences of Ariadne A and Ariadne B share a conserved sequence pattern (Figure 5): an acidic N-terminus, a complete Cys-rich domain of the RING1-IBR-RING2 structure, followed by a C-terminus, but with some variations, especially in the C-terminal sequence (Figure 5 and S4): compared with Ariadne A, most Ariadne B members contain another extremely conserved C-terminal motif ZF-RanBP (Zn-finger in Ran binding protein), which is found in RanBP2 (Ran binding protein 2) proteins, a 358 kD nucleoporin located on the cytoplasmic side of the nuclear pore complex and plays a role in nuclear protein import [30]. In order to see if this ZF-RanBP domain is present in other known Ariadne proteins or not, we searched the RanBP2 domain in the 16 ARIADNE family members in Arabidopsis (TAIR, http://www.arabidopsis.org/), and found four of them (AtARI8, AtARI14, AtARI15, AtARI16) contained a RanBP2 domain.

Two proteins in soybean belong to Helicase group. The full length of each protein is over 1700 amino acid residues. There are three additional typical domains in Helicases including DEAD, C-terminal Helicase domain, and HA2 (Helicase associated domain), which might be involved in RNA binding. These domains are separate by about 750 amino acid residues from the RBR supra-domain, which indicate that these proteins may be involved in the functions that need the participation of RNAs.

Soybean genome contains fourteen Plant II genes. These genes are further classified into three groups: Plant IIA, Plant IIB and Plant IIC (Figure 3 and Table 1). As shown in Figure 5, a RVT domain (PF00078) is present in the N-terminal of Plant IIA/B proteins; however, the proteins of Plant IIC have no obvious additional characteristic protein architecture in addition to the RBR domain.

### Exon-intron Structures of the Soybean RBR Genomic Sequences

**Figure 6:**
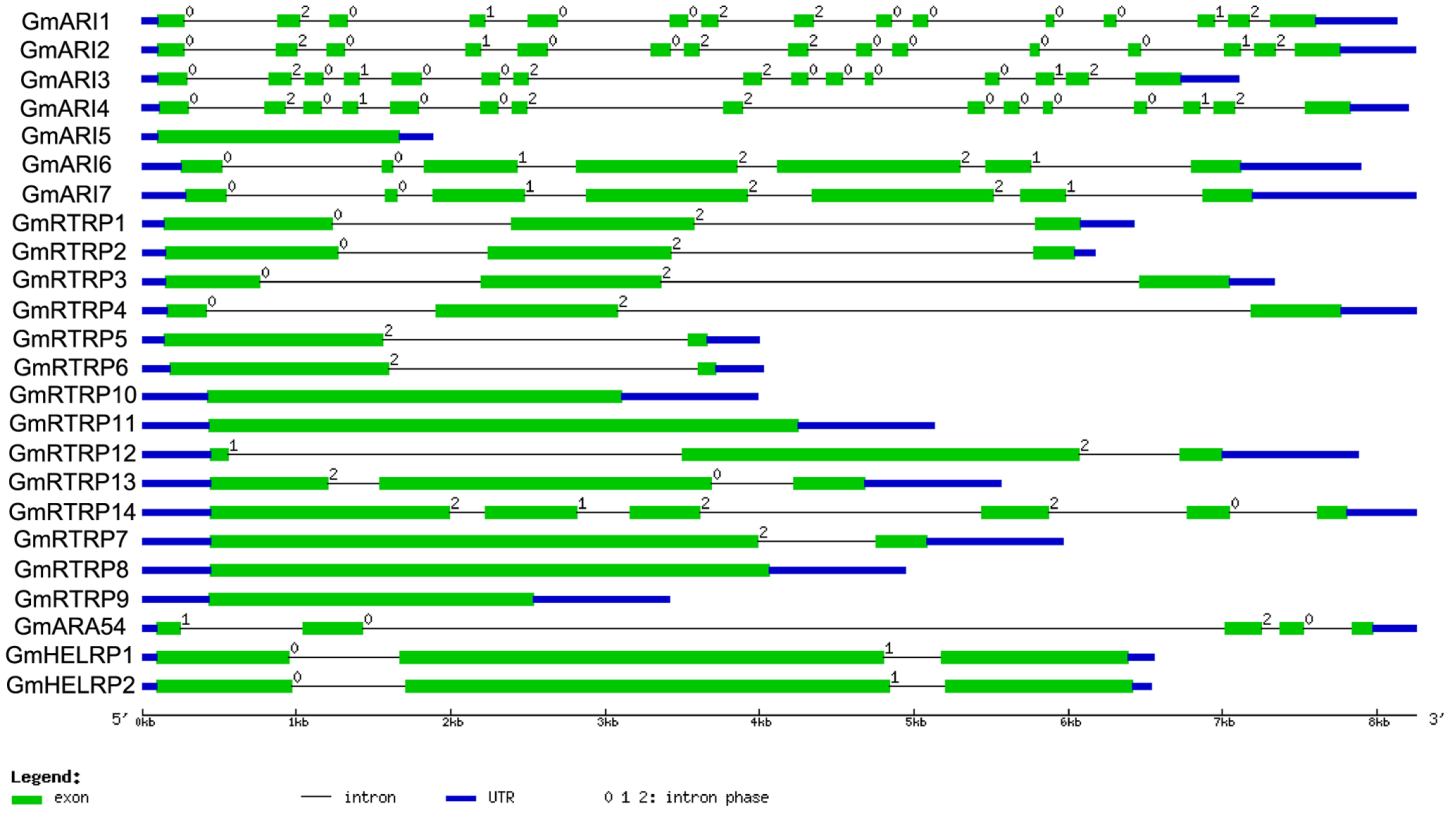
Intron/exon structures of RBR genes in *Glycine max*. Gene structures were drawn using online tool GSDS. As shown in the legend, green boxes stand for exons, black lines stand for introns, blue boxes are UTR regions.

We analyzed the genomic structures of soybean RBR genes including the number and length of introns/exons (Figure 6). As reflected by the diversity of these RBR proteins, the genomic structures are quite different between different subfamilies or groups: the number of introns varies from zero (Plant IIC) to fourteen (Ariadne B), and the length of the introns varies from ∼80 bp to ∼5.5 kb. The number of exons between groups also varies a lot: Ariadne B contains the largest number of exons (15 exons), while there are only two exons in *GmRTRP5* gene, and the length of the exons is between 48 bp and 3127 bp. Therefore, the exon/intron structures of soybean RBR genomic sequences were different between subfamilies and correlated with the protein sequence architecture and phylogenetic analysis. However, within each subfamily/group, the RBR genes share similar intron/exon architectures (Figure 6).

The soybean *ARA54* gene has a striking feature of a long intron. As shown in Figure 6, the length of the second intron is more than 5 kb. Its orthologous genes in *Arabidopsis thaliana*, *M. truncatula* and *P. vulgaris* have a shorter corresponding intron than *GmARA54*. To explore what had happened about the *GmARA54* gene, ∼17 kb length genomic sequence surrounding the predicted *GmARA54* gene was analyzed with FGENESH (http://linux1.softberry.com/berry.phtml) and GENSCAN [31]. We identified two longest predicted CDS sequences in this 17 kb region, which encoded two proteins of 494 and 1266 amino acid residues, respectively. These two proteins were analyzed using Pfam and SMART, as expected, the shorter protein shares identical sequence with the annotated soybean ARA54 protein sequence from Phytozome, containing the same RBR supra-domain and a N-terminal RWD domain. Surprisingly, the longer CDS encodes a putative Ty1-copia-type LTR retrotransposon protein and is located in the intron of the putative GmARA54. Further analyses indicated that this Ty1-copia type retrotransposon might be nonfunctional due to the presence of a stop codon in the code region (Figure S5). The insertion of this retrotransposon did not interrupt the correct splicing site of this intron, which indicates it might not affect the expression of *GmARA54* gene, as supported by the existence of the EST sequence of *GmARA54*.

### Sub-cellular Localization of Soybean RBR Proteins

**Figure 7:**
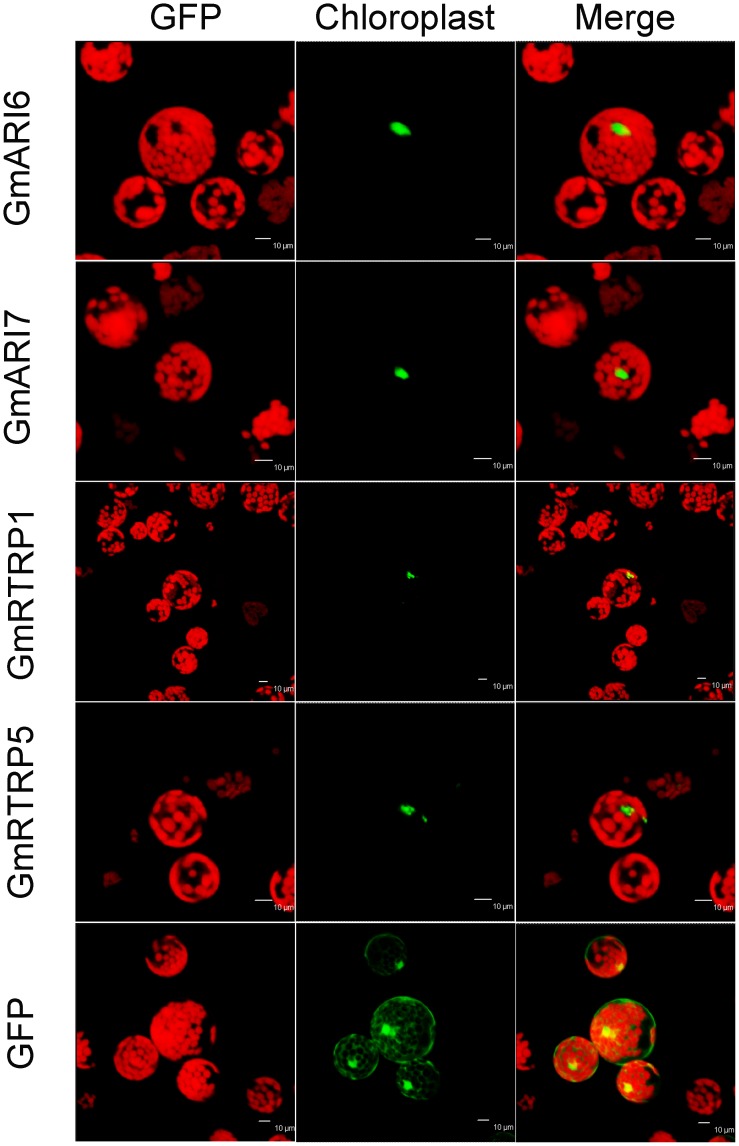
Nuclear localization of the soybean RBR proteins. RBR-GFP fusion proteins were transiently expressed in Arabidopsis mesophyll protoplasts and analyzed by confocal microscopy. Left: Fluorescent images of GFP; Middle: Chloroplast fluorescence; Right: Merge.

To investigate the potential sub-cellular sites of these soybean RBR proteins, their possible sub-cellular locations were first predicted using WOLF PSORT [32] and BaCelLo [33]. The prediction suggested that most of these soybean RBR proteins might be localized in nucleus (Table 1). Next, we searched for different signal peptides, but the soybean RBR proteins carry neither a signal peptide nor a trans-membrane domain. To experimentally confirm the sub-cellular locations of these proteins, GFP-fusion of four genes were transiently expressed in *Arabidopsis* mesophyll protoplast by PEG-mediated transformation. As shown in Figure 7, the GFP-fusion proteins of these four soybean RBR proteins, GmARI6, GmARI7, GmRTRP1 and GmRTRP5, were located in the nucleus, which agreed with the prediction.

### Expression Patterns of Soybean RBR Genes

**Figure 8:**
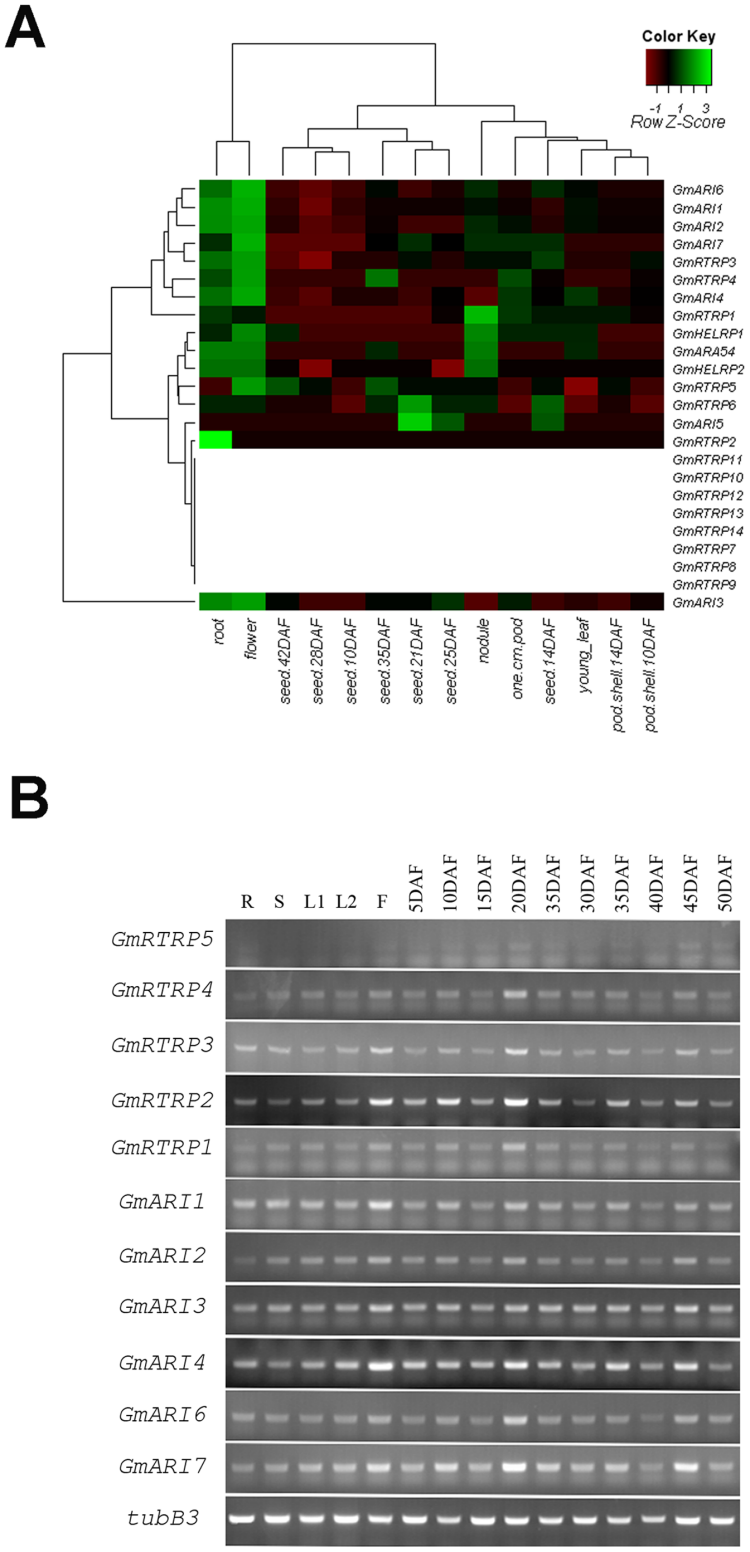
The expression patterns of soybean RBR genes. A. Heatmap of soybean RBR gene expressions in 14 tissues. RPKM normalized log2-transformed transcription counts data and microarray data for the soybean RBR genes are obtained from SoyBase (Table S4) and PLEXdb, respectively. Heatmaps were plotted using R. B. Semi-quantitative PCR analyses of soybean RBR genes expression patterns. R root, S stem, L1 and L2 Leaf, F flower, 5 DAF (day after flowering) ∼50 DAF, different developmental stages of pods.

To explore the expression patterns of these soybean RBR genes, BLASTN was performed in the NCBI GEO (http://www.ncbi.nlm.nih.gov/geo/) and Soybean Full-Length cDNA Database (http://rsoy.psc.riken.jp/). As summarized in Table 1, no ESTs were identified for ten of these genes, and seven of which seem to have incomplete protein sequences, which indicating these might be pseudogenes. Transcript abundance for soybean RBR genes was analyzed using the data from SoySeq (http://soybase.org/soyseq/, Table S4). As shown in Figure 8A, fifteen genes are broadly expressed with a relatively low level in soybean tissues, while *GmRTRP2* is only detected in roots. Most genes seem to be expressed at a higher level in roots and flowers. The seven putative pseudogenes showed no expression in the RNA-Seq data (Figure 8A).

To experimentally determine the tissue-specific expression pattern of soybean RBR genes, semi-quantitative PCR was performed using RNA isolated from roots, leaves, stems, flowers, and seeds from different development stages (Figure 8B). All eleven cloned RBR genes expressed in all five tissues and throughout the process of development of soybean seed, and many genes had a higher level in flowers, which agrees with the RNA-Seq data (Figure 8).

**Figure 9:**
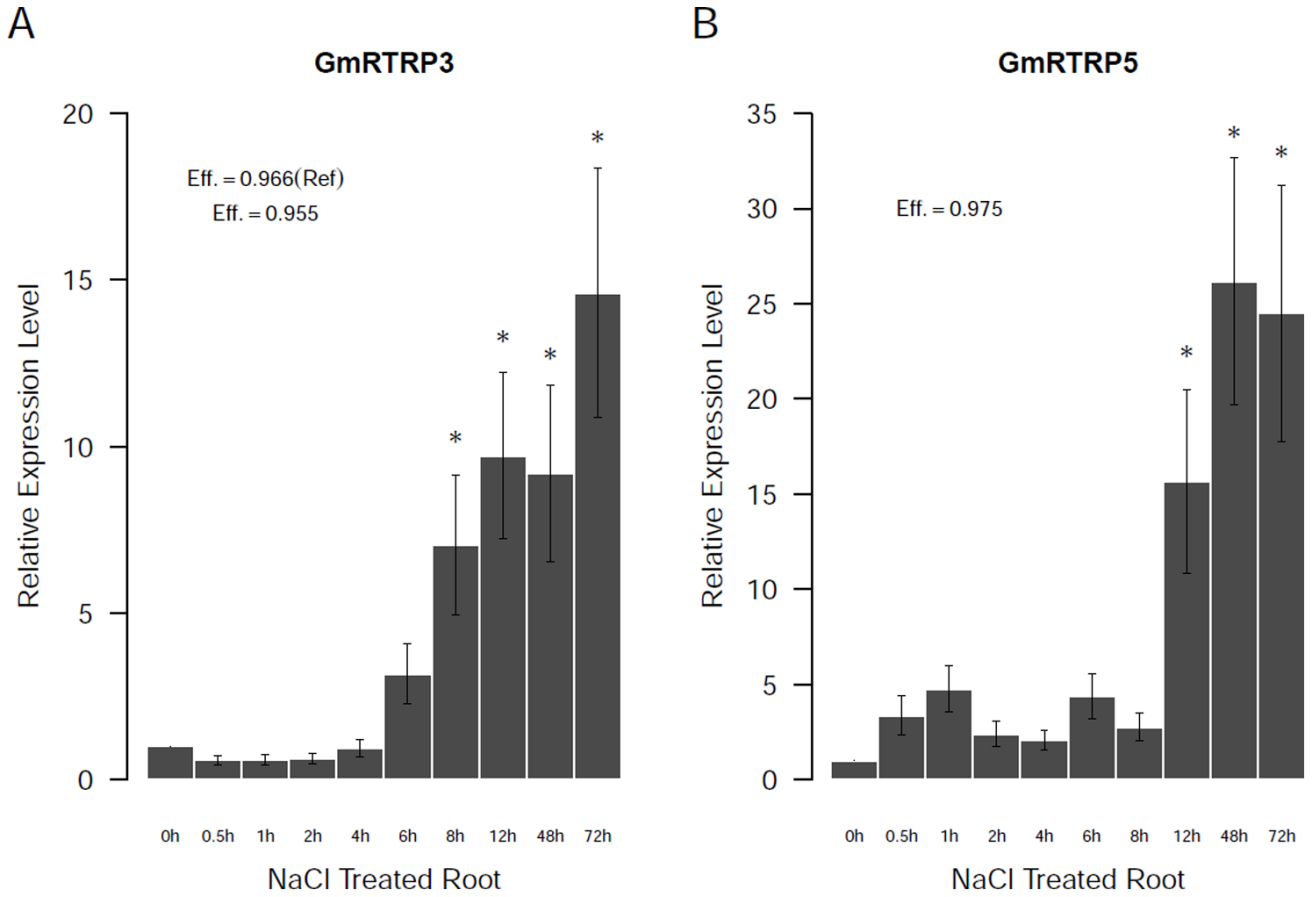
The relative gene expression of two soybean RBR genes in response to salt stress. Asterisks indicate the relative expression at these time points were statistically different from the 0(*p*<0.05, t-test).

To further investigate the possible functions of soybean RBR genes, the expression of Plant II genes in response to simulated salt stress were investigated by qRT-PCR analysis. The results indicate that the expression of *GmRTRTP3* and *GmRTRP5* gene are induced by NaCl treatment (Figure 9).

## Discussion

RBR proteins have been found in all eukaryotic organisms whose sequence data are available, and accumulating evidence suggests that RBR proteins play critical roles in many cellular processes. However, there are few reports on the detailed characterization and functions of RBR genes in plant, especially in legume. In this report, the genome-wide RBR genes of soybean were identified and characterized in details, which would be helpful to further study the function of RBR genes in this important crop.

Twenty-four genes encoding RBR proteins were identified in *G. max* genome, most of which contain a complete and conservative RBR supra-domain, including the highly conserved Cys and His residues, which were critical for Zn^2+^ binding and the senior protein structure. Previous studies on RBR proteins suggested that mutations in this domain could disrupt their senior structure and function [17], [19], [34], [35], for example, the PARKIN protein with the C332S or C365S mutations in IBR domain adopt an unfolded structure [17]. Furthermore, between the extremely conservative Cys and His residues, there are many other conserved residues sites, and some studies showed that these sites were also essential for the protein senior structures and functions [17], [35].

The results of phylogenetic analyses of 650 RBR protein sequences from 32 plant species had classified soybean RBR proteins into similar subfamilies as previous reports [12] based on the corresponding RBR domain sequences homology (Figure 2). However, we noticed that two subfamilies, Ariadne and Plant II [12], were shown to be more heterogeneous between the subgroups, the sequences of different subgroups contained quite different protein domains or motifs. Of these protein subfamilies, five subgroups (Ariadne A, Ariadne B, ARA54, Plant IIA and Plant IIB) were found to include new members from Algae, Bryophytes and Lycophyta. Previous research have revealed that RBR genes of Helicase subfamily were present in animal [11], but not in lower plants, these results suggested that RBR proteins might originate before the divergence of Algae and rest of plants, and were quite conserved across the evolution of viridiplantae. Whereas, the subfamilies Plant IIC and Helicase were only present in Angiosperm suggested that these two families might have experienced lost in the progress of plant evolution.

RBR gene expansion caused by block duplication or tandem duplication has been observed in the Ariadne B and Plant IIC genes in soybean, and also in *Medicago truncatula* and *Phaseolus vulgaris*. Evidence for one or two whole genome duplication (WGD) is found in the rice genome [36], whereas two or three rounds of WGD are apparent in poplar, Arabidopsis [37]–[39]. Previous study using synonymous distances among gene pairs within species and soybean genome sequence data has found evidences for large scale duplications in the legumes including *Glycine max* and *Medicago truncatula*, and that soybean is known to have undergone two rounds of polyploidy events [39]–[42]. It is reasonable to hypothesize that these paralogues genes arose from a recent genome duplication event. An alternative mechanism to generate new gene copies is retroposition, which might generate intronless pseudogenes. Several intron-less RBR genes might arise from retroposition, and these pseudogenes have neither ESTs nor expression (Table 1). In *Arabidopsis thaliana*, three Ariadne genes (*AtARI3/4* and *AtARI6*) have been identified as intronless genes generated by retroposition [43].

Although the RBR proteins contain extremely conservative Cys, His and some other residues, different groups contain quite different conserved non-RBR domain architectures (Figure 5). These different protein domains might account for the diversification of RBR proteins, and particularly, to some extent might determine the specifications of RBR protein functions. The research on the *Parkin* gene show that the N-terminal Unique Parkin domain (UPD, also annotated as RING0 could bury the catalytic Cys site through its interaction with RING2 domain [44]. Similarly, Duda and colleagues proved that the N-terminal Ariadne domain also could mask the catalytic Cys from RING2 to block E3 ligase activity [45]. The structure and biochemical studies suggest that significant conformational changes are essential for this RBR proteins activation, and any mutation on the conserved residues of RBR and non-RBR domains could lead to the disorder of senior structure and lose of their function.

The exon/intron structure of soybean RBR gene pairs shows high conservation within the same subfamily, consistent with a high degree of position and phase conservation found more broadly across legumes. However, the exon/intron structures of soybean RBR genomic sequences were different between subfamilies and correlated with the protein sequence architecture and phylogenetic analysis. Meanwhile, mutations were also present: a retrotransposon insertion was identified in the intron of *GmARA54* gene. Genome sequence of the palaeopolyploid soybean indicates that long terminal repeat (LTR) retrotransposons are the most abundant class of transposable elements [42]. Therefore, the similarity of these RBR sequences (conserved RBR domain) indicates that those genes might arise from the same ancient genes and share similar functions as E3 ligases, which combine with the ubiquitin-containing E2 enzymes, to recognize the target protein that is to be ubiquinated. On the other hand, vast structural diversification has occurred in parallel with sequence diversification and the emergence of RBR subfamilies, and previous extensive searches in animals demonstrated this phenomenon [8], [11], [46]. These diverse domains in RBR proteins might play critical roles in protein interactions and regulations involving in different cellular processes. The functional significance of the domains within RBR proteins and their interaction partners are not clear, which is certainly an urgent research area in the future. For example, it would be of interest to determine what role the RVT domain of the Plant IIA/B proteins plays.

The conserved domain and broad gene expression pattern (Figure 8B) of soybean RBR members agrees with previous study in *Arabidopsis thaliana* and *Oryza sativa* [12], which indicate that they might have some conserved molecular functions as E3 ligases. However, the variation in RBR members indicates the diversity of the RBR genes. Two soybean Plant II genes were found responsive to salt tress, indicating they might be involved in soybean response to abiotic stresses. Further functional analysis of these two genes would help understand the functions of RBR genes in plants.

## Supporting Information

Figure S1 **Phylogenetic analysis of 650 plant RBR protein sequences.** *Aquilegia coerulea* (Aco), *Arabidopsis lyrata* (Al), *Arabidopsis thaliana* (At), *Brachypodium distachyon* (Bd), *Brassica rapa* (Bra), *Capsella rubella* (Cru), *Carica papaya* (Cpa), *Chlamydomonas reinhardtii* (Cre), *Citrus clementina* (Ccl), *Citrus sinensis* (Csi), *Cucumis sativus* (Csa), *Eucalyptus grandis* (Eg), *Glycine max* (Gm), *Linum usitatissimum* (Lus), *Malus domestica* (Mdo), *Manihot esculenta* (Mes), *Medicago truncatula* (Mt), *Mimulus guttatus* (Mg), *Oryza sativa* (Os), *Phaseolus vulgaris* (Pvu), *Physcomitrella patens* (Ppa), *Populus trichocarpa* (Cru), *Prunus persica* (Ppe), *Ricinus communis* (Rco), *Selaginella moellendorffii* (Smo), *Setara italic* (Si), *Sorghum bicolor* (Sb), *Thellungiella halophila* (Th), *Vitis vinifera* (Vvi), *Volvox carteri* (Vc), *Zea mays* (Zm).
(TIF)

Figure S2 **Examples of the genome tandem duplication and block duplication of soybean RBR gene.** A. Block duplication of Ariadne subfamily. B. tandem duplication of Plant II subfamily. Synteny plot were performed in Plaza. The RBR genes are indicated with red box.
(TIF)

Figure S3 **Conservation and diversity of the RBR domains.** The schematic representation of the motif in RBR domain is elucidated by MEME. The first and last conserved Cys residues of RBR domain are marked by asterisks, while asterisks with a superscript number indicate responding conserved Cys residues site. RING1, IBR and RING2 domian are indicated with solid lines. The height of a letter in the Logo indicates its relative frequency at the given position (x -axis) in the motif.
(TIF)

Figure S4 **Conserved N- and C- terminus of Ariadne A and Ariadne B subfamilies protein sequences elucidated by MEME.** (A–C) N-terminus of Ariadne A subfamily proteins. (D, E) N-terminus of Ariadne B subfamily proteins. (F–I) C-terminus of Ariadne A subfamily proteins. (J–N) C-terminus of Ariadne B subfamily proteins. The height of a letter in the Logo indicates its relative frequency at the given position (x -axis) in the motif.
(TIF)

Figure S5 **Structure of the full-length ARA54 and Ty1-copia type retrotransposon element.** The exons of predicted GmARA54 gene are depicted as dark gray arrows, LTRs (long terminal repeat) are depicted as light gray arrows, the ORF is represented by a solid box, and positions of stop codons in reading frames are shown as vertical box. TSD (target-site duplication) are depicted with black box.
(TIF)

Table S1 Primers used for gene cloning and quantitative PCR in this study.
(DOCX)

Table S2 GenBank ID of cloned soybean RBR genes.
(DOCX)

Table S3 Real Time-PCR System.
(DOCX)

Table S4 RPKM normalized log2-transformed transcription counts data of the soybean RBR genes in 14 tissues [47].
(DOCX)

Additional file S1 Multi-alignment of 650 RBR protein sequences.
(TXT)
