# Supplementary figures and images for: Genome-Wide Identification and Characterization of RBR Ubiquitin Ligase Genes in Soybean
Source: PLoS One. 2014 Jan 28;9(1):e87282. doi: 10.1371/journal.pone.0087282 (PMC3904995; doi:10.1371/journal.pone.0087282)

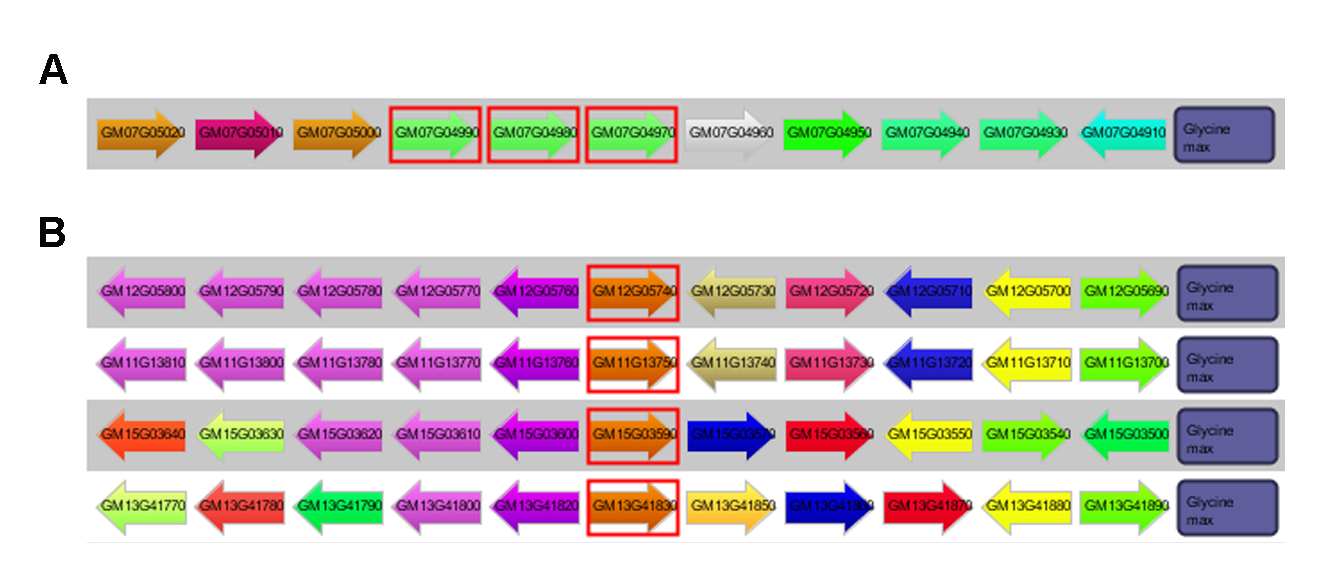

Supplement: Figure S2 — Examples of the genome tandem duplication and block duplication of soybean RBR gene. A. Block duplication of Ariadne subfamily. B. tandem duplication of Plant II subfamily. Synteny plot were performed in Plaza. The RBR genes are indicated with red box. (TIF) [file pone.0087282.s002.tif]

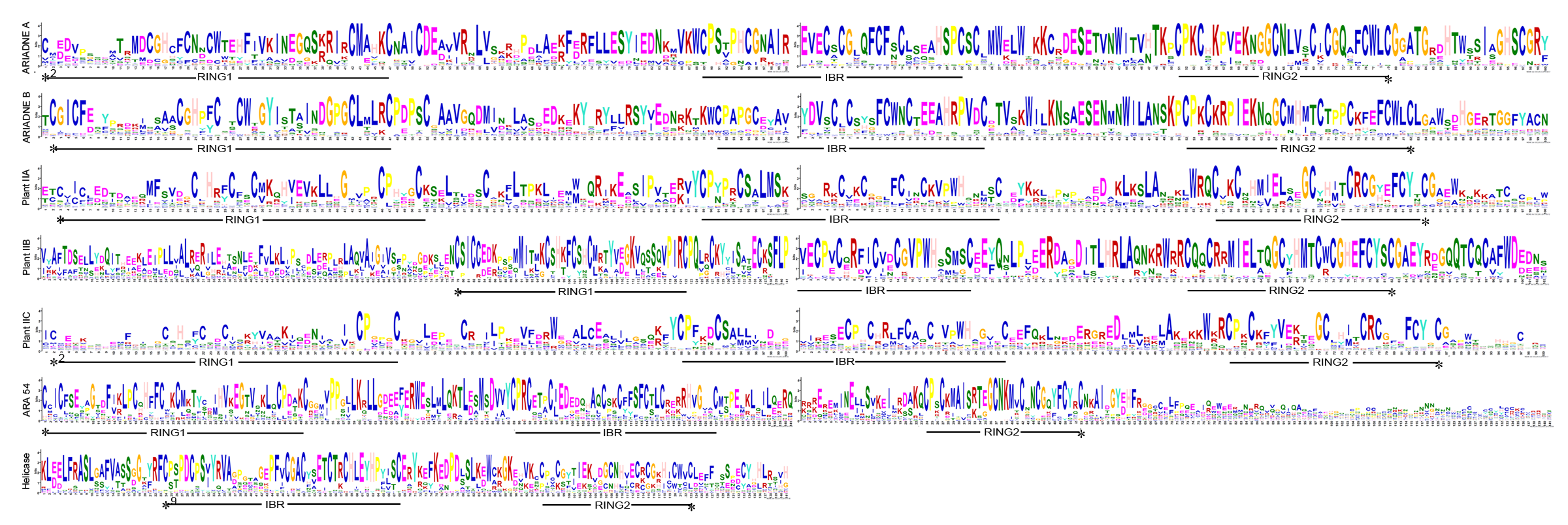

Supplement: Figure S3 — Conservation and diversity of the RBR domains. The schematic representation of the motif in RBR domain is elucidated by MEME. The first and last conserved Cys residues of RBR domain are marked by asterisks, while asterisks with a superscript number indicate responding conserved Cys residues site. RING1, IBR and RING2 domian are indicated with solid lines. The height of a letter in the Logo indicates its relative frequency at the given position (x -axis) in the motif. (TIF) [file pone.0087282.s003.tif]

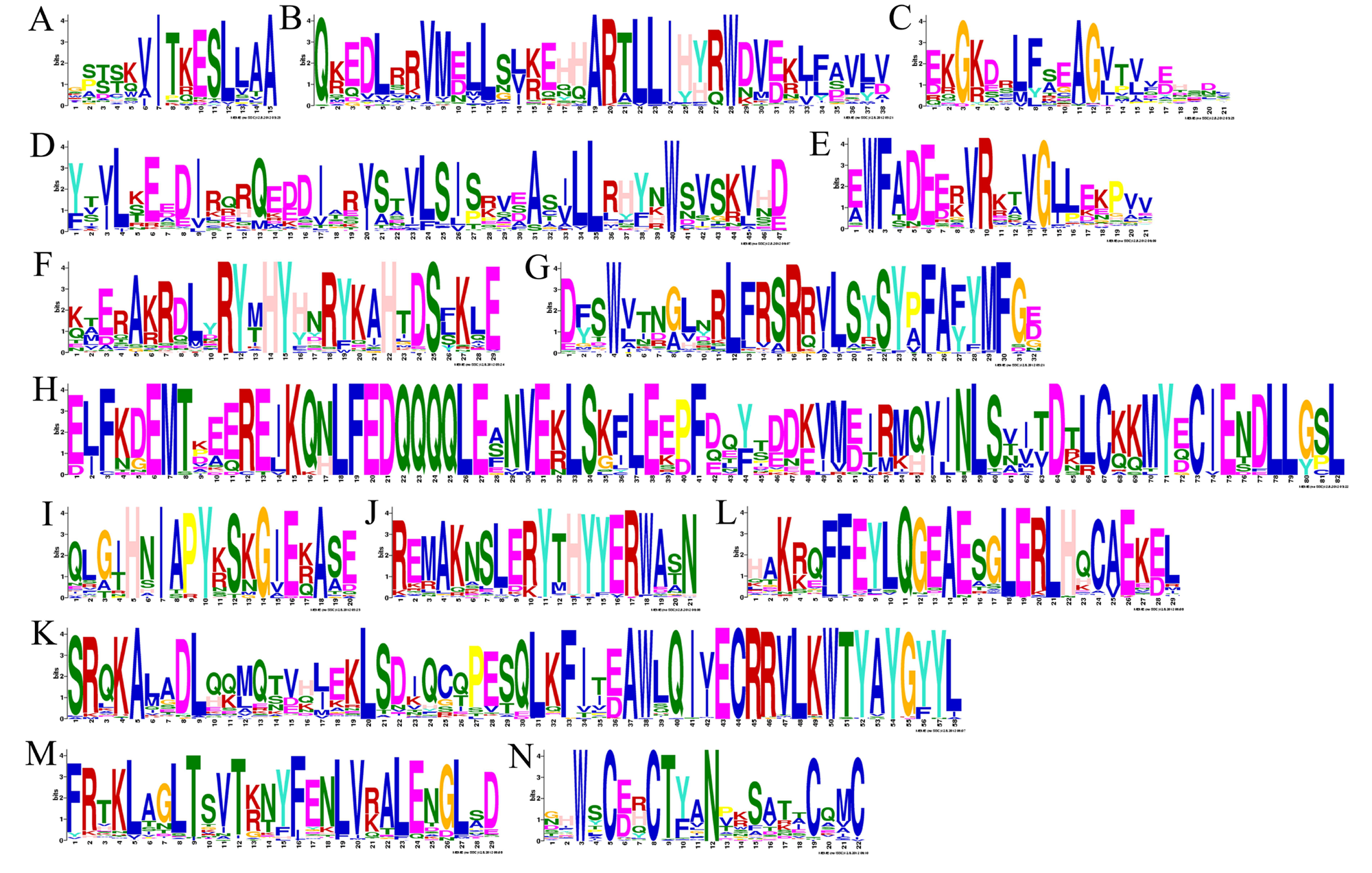

Supplement: Figure S4 — Conserved N- and C- terminus of Ariadne A and Ariadne B subfamilies protein sequences elucidated by MEME. (A–C) N-terminus of Ariadne A subfamily proteins. (D, E) N-terminus of Ariadne B subfamily proteins. (F–I) C-terminus of Ariadne A subfamily proteins. (J–N) C-terminus of Ariadne B subfamily proteins. The height of a letter in the Logo indicates its relative frequency at the given position (x -axis) in the motif. (TIF) [file pone.0087282.s004.tif]

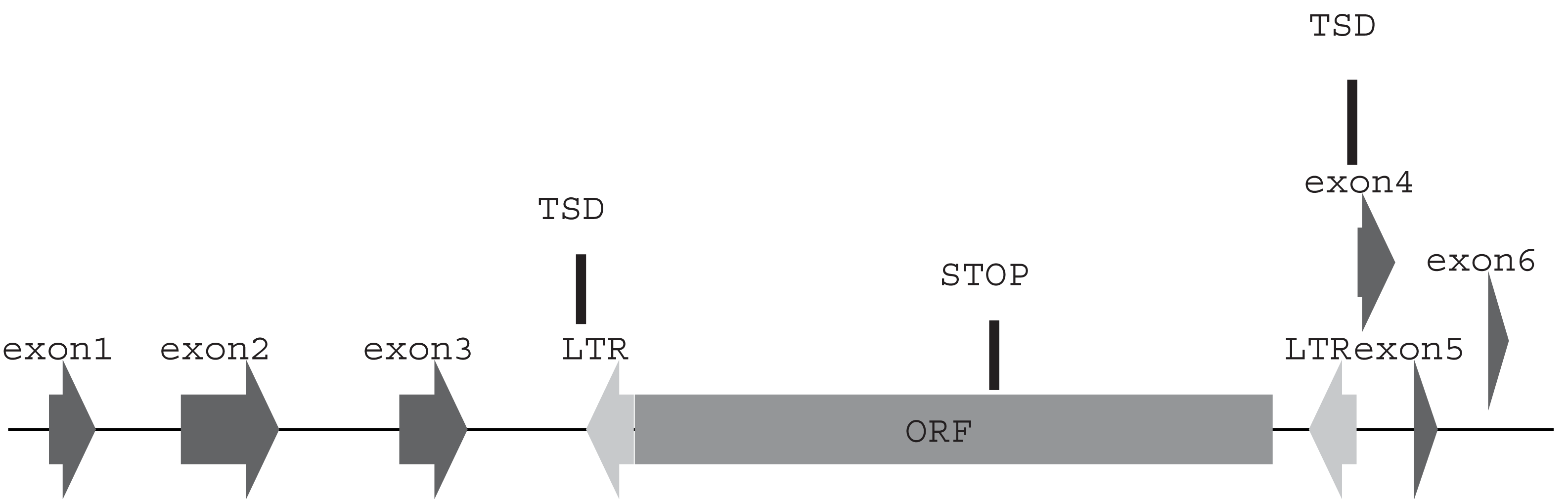

Supplement: Figure S5 — Structure of the full-length ARA54 and Ty1-copia type retrotransposon element. The exons of predicted GmARA54 gene are depicted as dark gray arrows, LTRs (long terminal repeat) are depicted as light gray arrows, the ORF is represented by a solid box, and positions of stop codons in reading frames are shown as vertical box. TSD (target-site duplication) are depicted with black box. (TIF) [file pone.0087282.s005.tif]
