# Supplementary material for: Genome-Wide Identification and Characterization of RBR Ubiquitin Ligase Genes in Soybean
Source: PLoS One. 2014 Jan 28;9(1):e87282. doi: 10.1371/journal.pone.0087282 (PMC3904995; doi:10.1371/journal.pone.0087282)
Supplement: Table S1 — Primers used for gene cloning and quantitative PCR in this study. (DOCX) [file pone.0087282.s006.docx]

**Table S1 Primers used for gene cloning and quantitative RT-PCR**

| **Gene cloning primers** | |
| --- | --- |
| GmARI3-F | 5' TTCCCCTCATTCTACCTTCCTTC 3' |
| GmARI3-R | 5' CCTGTTGCATAAACCCCATACG 3' |
| GmARI6-F | 5' ATCCCTAATTCTGCGATTCCATC 3' |
| GmARI6-R | 5' AGTTGTCCTCAAGGTAGCCATTT 3' |
| GmARI7-F | 5' TCGCCGGCGCTCTCGTCGGAATT 3' |
| GmARI7-R | 5' ACAGAAAGGTAGATGGCATAGGA 3' |
| GmHELRP1-F | 5' GATCGACGAATGCCACTCCC 3' |
| GmHELRP1-R | 5' GCACCAGACCCAAACAAAGC 3' |
| GmHELRP1-F1 | 5' CGTTGTTACTCCGAGTTACTGGC 3' |
| GmHELRP1-R1 | 5' TGGTACGCAAATGGCAATTTATA 3' |
| GmRTRP1-F | 5' CTCACCCGCGAACCTGAATAC 3' |
| GmRTRP1-R | 5' ATGAAACGGAAAATAAGGAGAAAGA 3' |
| GmRTRP2-F | 5' TCCGTCGCAGCTATCGAACAAC 3' |
| GmRTRP2-R | 5' GTAAAACCGTCAACCTGGTCAAGC 3' |
| GmRTRP3-F | 5' TTCTTTTGGGTTTAGCAGCCTC 3' |
| GmRTRP3-R | 5' CACAAACTCTTCCTGCATCCTTT 3' |
| GmRTRP4-F | 5' TTGTAGTCGAAGGGTTGAAGGAT 3' |
| GmRTRP4-R | 5' TTCTTCCCAACCACAAGTAAAATG 3' |
| GmRTRP5-F | 5' TTGGAGAAAGTGGTCAGTGGAGA 3' |
| GmRTRP5-R | 5' GGTGTTTGGGATGAGTTTAGGTAGT 3' |
| **Semi-quantitative RT-PCR primers** | |
| Tubulin B3 | 5' ATCTCATTCCCTTCCCTCGTCTG 3' |
|  | 5' ATCTGCTCGTCCACCTCCTTG 3' |
| GmARI6 | 5' CTCTGACTCACCTGTTCCTCCTG 3' |
|  | 5' CTGATTCATCTCGGCACTTCTTAGC 3' |
| GmARI7 | 5' GCAAGCGTAGGTGTAACAGAAGAAC 3' |
|  | 5' TCCAAAGGCAGCAAAGCAAAGC 3' |
| GmRTRP1 | 5' CGTCGTCTTCGTCGCTGAGTAG 3' |
|  | 5' ATTATCGCTAAGGTCGCAAATCGC 3' |
| GmRTRP2 | 5' GAACACAGCGAGGCGGAGAC 3' |
|  | 5' TCCCAACGAGCGACTTAGAAACC 3' |
| GmRTRP5 | 5' GAGGCTGTGATTGCTGCTGAAG 3' |
|  | 5' GGTGGTGTTTGGGATGAGTTTAGG 3' |
| GmRTRP3 | 5' TTATGCGGAGGAGTCTGTGGTTG 3' |
|  | 5' TGGCTGCTCAAGATCACTAGAAGG 3' |
| GmRTRP4 | 5' CAGCGAGGAGGAGATTGGTAAGG 3' |
|  | 5' CAGCAGTGTCGTAGAATGATTGAGC 3' |
| GmARI1 | 5' CTCCATTCTCCATTCTCCTCTTTGC 3' |
|  | 5' GTCGTCGTCGCTGTAGTAGTCC 3' |
| GmARI2 | 5' GATGGAGTCGGAGGATGATATGCC 3' |
|  | 5' TGGTGACGGAGTAGTATGCTTGC 3' |
| GmARI3 | 5' GACGACAACGCCGACGATTAC 3' |
|  | 5' GCCACAAGCAGTAGATTCAACCC 3' |
| GmARI4 | 5' CCTCTTCATTCCTTCCTTCACCTTC 3' |
|  | 5' GCCACAAGCAGCAGATTCAACC 3' |
| **Quantitative RT-PCR primers** | |
| GmRTRP3-F | 5' GCCAGAGGAGAGGGATGCTTC 3' |
| GmRTRP3-R | 5' CGACACTGCTGACAACGCTTC 3' |
| GmRTRP5-F | 5' GCGGACGAGAGGGATAAGGAG 3' |
| GmRTRP5-R | 5' GTAACAGAAGGCGTTTCCACACC 3' |
| TubB3-F | 5' TCATTCCCTTCCCTCGTCTGC 3’ |
| TubB3-R | 5' CCTCCTTGGTGCTCATCTTGC 3’ |
