# Supplementary material for: Genome-Wide Identification and Characterization of RBR Ubiquitin Ligase Genes in Soybean
Source: PLoS One. 2014 Jan 28;9(1):e87282. doi: 10.1371/journal.pone.0087282 (PMC3904995; doi:10.1371/journal.pone.0087282)
Supplement: Table S2 — GenBank ID of cloned soybean RBR genes. (DOCX) [file pone.0087282.s007.docx]

**Table S2 GenBank ID of cloned soybean RBR genes**

| **Gene name** | **GenBank ID** |
| --- | --- |
| GmHELRP1 | JX392389 |
| GmARI1 | JX392390 |
| GmARI2 | JX392391 |
| GmARI3 | JX392392 |
| GmARI7 | JX392393 |
| GmRTRP2 | JX392394 |
| GmRTRP3 | JX392395 |
| GmRTRP4 | JX392396 |
| GmRTRP5 | JX392397 |
