# Supplementary material for: Genome-Wide Identification and Characterization of RBR Ubiquitin Ligase Genes in Soybean
Source: PLoS One. 2014 Jan 28;9(1):e87282. doi: 10.1371/journal.pone.0087282 (PMC3904995; doi:10.1371/journal.pone.0087282)
Supplement: Table S3 — Real Time-PCR System. (DOCX) [file pone.0087282.s008.docx]

**Table S3 Real Time-PCR System**

| **Component** | | **Added Volume (*μ*l)** | **Finally Concentrion** |
| --- | --- | --- | --- |
| 10 × PCR Buffer | 100mM Tris-HCl, pH 8.5 | 2.0 | 1× |
|  | 1000 mM KCl |  |  |
|  | 25 mM MgCl_2_ |  |  |
|  | 1.0% Triton X-100 |  |  |
|  | 0.5 mg/ml BSA |  |  |
| Mg^2+^(25mM) | | 2.0 | 2.5mM |
| dNTP(2.5mM each) | | 1.6 | 0.8mM |
| BSA(10mg/ml) | | 0.1 | 0.05 mg/ml |
| 1/1000 SYBR Green Ⅰ | | 1.0 | 1/20000 |
| ROX Dye | | 0.1 | 1/200 |
| Glycerol | | 2.0 | 1:10(v/v) |
| Taq polymerase(5U/μl) | | 0.15 | 1.5U |
| Forward primer (50μM) | | 0.05 | 250 nM |
| Reverse primer (50μM) | | 0.05 | 250 nM |
| Template(cDNA/100) | | 5 |  |
| ddH_2_O | | 5.95 |  |
| **Total** | | **20** |  |
