# Supplementary material for: Genome-Wide Identification and Characterization of RBR Ubiquitin Ligase Genes in Soybean
Source: PLoS One. 2014 Jan 28;9(1):e87282. doi: 10.1371/journal.pone.0087282 (PMC3904995; doi:10.1371/journal.pone.0087282)
Supplement: Table S4 — RPKM normalized log2-transformed transcription counts data of the soybean RBR genes in 14 tissues [47]. (DOCX) [file pone.0087282.s009.docx]

**Table S4 RPKM normalized log2-transformed transcription counts data about the soybean RBR genes in 14 tissues [47].**

| **Gene Name** | **Locus** | **young leaf** | **flower** | **one cm pod** | **pod shell 10DAF** | **pod shell 14DAF** | **seed 10DAF** | **seed 14DAF** | **seed 21DAF** | **seed 25DAF** | **seed 28DAF** | **seed 35DAF** | **seed 42DAF** | **root** | **nodule** |
| --- | --- | --- | --- | --- | --- | --- | --- | --- | --- | --- | --- | --- | --- | --- | --- |
| GmRTRP7 | Glyma07g04970 | 0 | 0 | 0 | 0 | 0 | 0 | 0 | 0 | 0 | 0 | 0 | 0 | 0 | 0 |
| GmRTRP14 | Glyma07g04980 | 0 | 0 | 0 | 0 | 0 | 0 | 0 | 0 | 0 | 0 | 0 | 0 | 0 | 0 |
| GmRTRP8 | Glyma07g04990 | 0 | 0 | 0 | 0 | 0 | 0 | 0 | 0 | 0 | 0 | 0 | 0 | 0 | 0 |
| GmRTRP3 | Glyma09g02340 | 4 | 8 | 5 | 5 | 4 | 4 | 6 | 5 | 4 | 2 | 4 | 3 | 7 | 5 |
| GmRTRP5 | Glyma09g08670 | 0 | 4 | 1 | 1 | 2 | 1 | 2 | 2 | 2 | 2 | 3 | 3 | 1 | 2 |
| GmARA54 | Glyma09g33900 | 2 | 3 | 1 | 1 | 1 | 1 | 1 | 1 | 1 | 1 | 2 | 1 | 3 | 3 |
| GmARI7 | Glyma11g12920 | 4 | 9 | 6 | 4 | 4 | 3 | 6 | 6 | 5 | 3 | 5 | 3 | 6 | 6 |
| GmARI1 | Glyma11g13750 | 5 | 10 | 4 | 4 | 4 | 3 | 3 | 4 | 4 | 1 | 4 | 3 | 9 | 5 |
| GmRTRP1 | Glyma11g15820 | 5 | 5 | 6 | 4 | 5 | 2 | 5 | 2 | 4 | 2 | 2 | 2 | 6 | 10 |
| GmRTRP9 | Glyma11g23590 | 0 | 0 | 0 | 0 | 0 | 0 | 0 | 0 | 0 | 0 | 0 | 0 | 0 | 0 |
| GmRTRP10 | Glyma11g23850 | 0 | 0 | 0 | 0 | 0 | 0 | 0 | 0 | 0 | 0 | 0 | 0 | 0 | 0 |
| GmHELRP2 | Glyma11g37910 | 1 | 2 | 1 | 1 | 1 | 1 | 1 | 1 | 0 | 0 | 1 | 1 | 2 | 2 |
| GmARI5 | Glyma12g03030 | 0 | 0 | 0 | 0 | 0 | 0 | 1 | 2 | 1 | 0 | 0 | 0 | 0 | 0 |
| GmARI6 | Glyma12g05050 | 5 | 10 | 4 | 4 | 4 | 3 | 6 | 3 | 4 | 2 | 5 | 3 | 8 | 6 |
| GmARI2 | Glyma12g05740 | 6 | 12 | 6 | 5 | 5 | 3 | 4 | 3 | 3 | 2 | 5 | 4 | 11 | 7 |
| GmRTRP2 | Glyma12g07640 | 0 | 0 | 0 | 0 | 0 | 0 | 0 | 0 | 0 | 0 | 0 | 0 | 1 | 0 |
| GmARI4 | Glyma13g41830 | 8 | 12 | 8 | 6 | 5 | 5 | 6 | 4 | 6 | 3 | 5 | 4 | 10 | 3 |
| GmARI3 | Glyma15g03590 | 8 | 17 | 11 | 9 | 7 | 7 | 7 | 10 | 12 | 7 | 10 | 10 | 16 | 6 |
| GmRTRP4 | Glyma15g13240 | 5 | 10 | 8 | 6 | 5 | 5 | 6 | 5 | 5 | 5 | 9 | 5 | 8 | 5 |
| GmRTRP6 | Glyma15g20350 | 0 | 2 | 0 | 0 | 1 | 0 | 3 | 4 | 2 | 1 | 2 | 1 | 2 | 2 |
| GmRTRP11 | Glyma16g01530 | 0 | 0 | 0 | 0 | 0 | 0 | 0 | 0 | 0 | 0 | 0 | 0 | 0 | 0 |
| GmHELRP1 | Glyma18g01820 | 2 | 3 | 2 | 1 | 1 | 1 | 2 | 1 | 1 | 1 | 1 | 2 | 2 | 3 |
| GmRTRP12 | Glyma18g07170 | 0 | 0 | 0 | 0 | 0 | 0 | 0 | 0 | 0 | 0 | 0 | 0 | 0 | 0 |
| GmRTRP13 | Glyma18g07180 | 0 | 0 | 0 | 0 | 0 | 0 | 0 | 0 | 0 | 0 | 0 | 0 | 0 | 0 |
